# Inhibitory effect of LY 255283 on the synthesis of leukotriene B_4_ and thromboxane A_2_ in human peripheral blood polymorphonuclear leukocytes and monocytes

**DOI:** 10.1155/S0962935193000572

**Published:** 1993

**Authors:** M. Uğur, M. Melli

**Affiliations:** Department of Pharmacology Medical Faculty of Ankara University Sihhiye Ankara 06100 Turkey

## Abstract

LY 255283 [(1-(5-ethyl-2-hydroxy-4-(6-methyl-6-)1H-tetrazol-5-yl)-heptyloxy) phenyl)ethanone], a specific leukotriene B_4_ (LTB_4_) receptor antagonist, inhibited the production of LTB_4_ in human peripheral blood polymorphonuclear leukocytes (PMNL) and in monocytes activated by calcium ionophore A23187. In human monocytes activated by ionophore it inhibited also the production of thromboxane B_2_ (TXB_2_). The effect of LY 255283 on 5-lipoxygenase (5-LO) and LTA_4_ hydrolase activities which catalyse the production of LTB_4_ and LTA_4_ has not been studied yet. It is thought that LY 255283 may inhibit the production of LTB_4_ and TXA_2_ by antagonising the effect of ionophore-induced LTB_4_ on 5-lipoxygenase and cyclooxygenase in human peripheral blood PMNL and monocytes.


Mediators of Inflammation 2, 407–409 (1993)

### Inhibitory effect of LY 255283 on the synthesis of leukotriene $B_4$ and thromboxane $A_2$ in human peripheral blood polymorphonuclear leukocytes and monocytes

M. Uğur and M. Melli[CA]

Department of Pharmacology, Medical Faculty of Ankara University, Sihhiye 06100, Ankara, Turkey

[CA] Corresponding Author

**LY 255283 [(1-(5-ethyl-2-hydroxy-4-(6-methyl-6-)1H-tetrazol-5-yl)-heptyloxy) phenyl)ethanone], a specific leukotriene $B_4$ ($LTB_4$) receptor antagonist, inhibited the production of $LTB_4$ in human peripheral blood polymorphonuclear leukocytes (PMNL) and in monocytes activated by calcium ionophore A23187. In human monocytes activated by ionophore it inhibited also the production of thromboxane $B_2$ ($TXB_2$). The effect of LY 255283 on 5-lipoxygenase (5-LO) and $LTA_4$ hydrolase activities which catalyse the production of $LTB_4$ and $LTA_4$ has not been studied yet. It is thought that LY 255283 may inhibit the production of $LTB_4$ and $TXA_2$ by antagonising the effect of ionophore-induced $LTB_4$ on 5-lipoxygenase and cyclooxygenase in human peripheral blood PMNL and monocytes.**

**Key words:** Human monocytes, Human polymorphonuclear leukocytes (PMNL), Leukotriene $B_4$ ($LTB_4$), LY 255283, Thromboxane $B_2$ ($TXB_2$)

### Introduction

$LTB_4$, a 5-lipoxygenase metabolite of arachidonic acid (AA), is an inflammatory mediator released from immunoinflammatory cells, especially from neutrophils and monocytes. A variety of stimuli, including calcium ionophores, particulate (zymosan) and soluble stimuli (N-formyl-methionyl-leucyl-phenylalanine) and complement component C5a[1,2] can elicit the synthesis of $LTB_4$ from neutrophil and monocytes.

It has been shown that $LTB_4$ stimulates various neutrophil functions such as chemotaxis, adherence, aggregation and degranulation.[3] $LTB_4$ also has some effects on the function of monocytes.[4]

Increased levels of $LTB_4$ have been shown during various immunoinflammatory conditions.[5,6] Since $LTB_4$ is accepted as an inflammatory mediator and the level of $LTB_4$ is increased during various immunoinflammatory conditions, several 5-lipoxygenase inhibitors and $LTB_4$ receptor antagonists are developed for the purpose of obtaining an effective antiinflammatory drug.[7]

Recently, McDonald *et al.* have shown that exogenously applied $LTB_4$ activates the human neutrophil 5-lipoxygenase and causes the overproduction of $LTB_4$.[8] During our ongoing studies with $LTB_4$ receptor antagonists, we have observed that LY 2455283, a specific $LTB_4$ antagonist,[9] can inhibit calcium ionophore A23187-induced $LTB_4$ synthesis in human neutrophils, monocytes and $TXB_2$ synthesis in monocytes.

### Materials and Methods

*Cell separation:* Citrated blood was collected by venipuncture from healthy volunteers who had abstained from any drugs for at least 1 week before the sampling. A method described by Dewar was used for the isolation of human PMNL and monocytes.[10] Briefly, platelet rich plasma was discarded after the centrifugation of whole blood. Mononuclear cells and PMNL were separated by centrifugation on Ficoll–Paque cushions. The PMNL were separated from erythrocytes by dextran sedimentation and $NH_4Cl$ lysis. After the isolation procedure, the purity of PMNL was $>98\%$ and viability determined by Trypan blue exclusion $>95\%$. Monocytes were further purified by adherence. 1.5 ml portions of the mononuclear cell suspension ($2 \times 10^6$ cells/ml) were layered onto 30 mm plastic tissue culture dishes (Greiner) and were incubated at 37°C under an atmosphere of 5% $CO_2$ and 95% air for 1 h. The nonadherent cells were removed and additional 1.5 ml portions of mononuclear cell suspension ($2 \times 10^6$ cells/ml) were added to each plate and the same incubation protocol was repeated. By quantitation of the adherent cells, it was determined that 85 to 90% of the adherent cells were monocytes.

*Cell incubation:* Incubations were performed at 37°C under an atmosphere of 5% $CO_2$ and 95% air. PMNL ($4 \times 10^6$ cells/ml) were suspended in Dulbecco's phosphate buffered saline containing

© 1993 Rapid Communications of Oxford Ltd

$Ca^{2+}/Mg^{2+}$ ($DPBS^{++}$) (composition in g/l NaCl 8, KCl 0.2, $Na_2HPO_4$ 1.15, $KH_2PO_4$ 0.2, $MgCl_2.6H_2O$ 0.1 and $CaCl_2$ 0.1) in tissue culture tubes (Greiner). After 10 min preincubation with LY 255283, PMNL were stimulated with 1 $\mu$M A23187. After an incubation period of 5 min, the cell suspension was centrifuged and the supernatant was stored at $-70°C$ until the quantitation of $LTB_4$ and $TXB_2$.

The monocyte monolayers were washed and fresh Dulbecco's modified eagle medium (DMEM) containing 5% foetal calf serum was added. After 10 min preincubation with LY 255283 ($10^{-6}$ M), monolayers were stimulated with 1 $\mu$M A23187. The supernatants were decanted, centrifuged and stored at $-70°C$.

*Enzyme immunoassay experiments:* $LTB_4$ and $TXB_2$ were measured by enzyme immunoassay (EIA).[11] The assay was performed in a total volume of 150 $\mu$l with each of the following components being added in a 50 $\mu$l volume: standards or biological samples, enzymatic tracer and specific antiserum. After overnight incubation at room temperature, the plates were washed and 200 $\mu$l Ellman's reagent was dispensed into each well. After 1–2 h, the absorbance at 405 nm of each well was measured. A standard curve from 15 pg/ml to 1 ng/ml was used in order to evaluate the concentrations of $LTB_4$ and $TXB_2$. Results are calculated in terms of per cent of B/Bo.

*Chemicals:* Calcium ionophore A23187 was purchased from Sigma Chemical Company (St Louis, MO). Monoclonal anti-rabbit IgG, standards and enzymatic tracers of $LTB_4$ and $TXB_2$ and antibody to $LTB_4$ were purchased from Cayman Chemical Company (Ann Arbor, MI). DMEM was purchased from Gibco BRL (Middlesex, UK). LY 255283 and antibody to $TXB_2$ were kindly provided by Thomas L. Jeatran (Lilly Research Laboratories, Indianapolis), and Professor GianCarlo Folco (Institute of Pharmacological Sciences, University of Milan) respectively. A23187 and LY 255283 were dissolved in absolute ethanol and dimethylsulfoxide respectively and were diluted with $DPBS^{++}$.

*Statistics:* The results are expressed in ng/$10^6$ cells and ng/dish for PMNL and monocytes respectively. The statistical assessment of groups was made by using a nonparametric method, Mann–Whitney–Wilcoxon test. The minimal level of significance was considered as $p < 0.05$.

### Results

Calcium ionophore A23187 ($10^{-6}$ M) elicited an increase in the synthesis of $LTB_4$ and $TXB_2$ in human PMNL and monocytes. This increase was more prominent on the synthesis of $LTB_4$, especially in monocytes (Figs 1 and 2).

$LTB_4$ receptor antagonist LY 255283, at the concentration of $10^{-6}$ M, significantly inhibited the ionophore-induced $LTB_4$ synthesis in both cell types (Figs 1 and 2). In preliminary experiments, LY 255283 (ranging from $10^{-9}$ to $10^{-6}$ M) caused a dose-dependent inhibitory effect on $LTB_4$ release (data not shown). LY 255283 also inhibited the synthesis of $TXB_2$, the stable metabolite of $TXA_2$ in monocytes (Fig. 2). However, LY 255283 did not significantly inhibit the synthesis of $TXB_2$ in PMNL (Fig. 1).

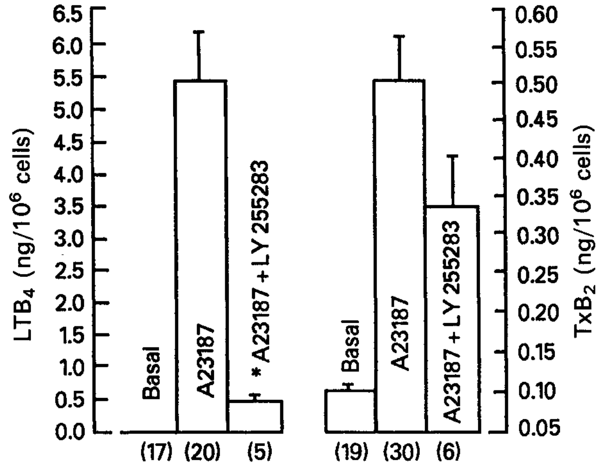

FIG. 1. Inhibition by LY 255283 ($10^{-6}$ M) of the A23187-induced ($10^{-6}$ M) increase of $LTB_4$ (*$p < 0.0007$) in human PMNL. ( ) number of experiments.

### Discussion

It is well known that a variety of stimuli, including the calcium ionophore A23187, can elicit an increase in the synthesis of cyclooxygenase (prostaglandin $E_2$, prostacylin, $TXA_2$) and lipoxygenase (mainly $LTB_4$) metabolites of AA in human

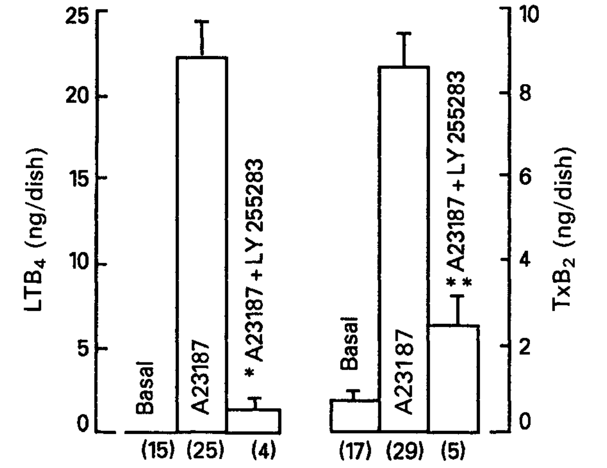

FIG. 2. Inhibition by LY 255283 ($10^{-6}$ M) of the A23187-induced ($10^{-6}$ M) increase of $LTB_4$ (*$p < 0.001$) and $TXB_2$ (**$p < 0.003$) in human monocytes. ( ) number of experiments.

PMNL and monocytes.[1,2] In the present study, calcium ionophore A23187, produced an increase in the synthesis of $LTB_4$ and $TXB_2$ in these cells. The ionophore-induced synthesis of $LTB_4$ was more prominent than that of $TXB_2$, a finding which supports previous observation of Moroney *et al.*[12]

$LTB_4$ is also known as one of the activators of human PMNL and monocytes. It is therefore expected that $LTB_4$ may activate its own synthesis in these cells. McDonald *et al.*[8] have described that 14,15-dideuterio-$LTB_4$ (D2-$LTB_4$) activates 5-lipoxygenase in PMNL and elicits an increase of $LTB_4$ synthesis. In this study, increased synthesis of $LTB_4$ by D2-$LTB_4$ in PMNL occurred when the cells were incubated with exogenous AA. It is well known that synthesis of $LTB_4$ from endogenous AA results from the activation of two independent calcium-dependent events. First, the release of AA from membrane phospholipids and second, the activation of the 5-LO.[13] In the present study, calcium ionophore A23187 increases $Ca^{2+}$ influx as well as intracellular $Ca^{2+}$ and therefore causes the release of AA from membrane phospholipids and activation of 5-LO.

Another interesting facet of the present study is that LY 255283 causes a decrease in the level of $TXB_2$ in monocytes. $LTB_4$ increases $PGE_2$ and $TXA_2$ synthesis in rat peritoneal macrophages elicited by carrageenin injection.[14] The results of the present study indicate that LY 255283 inhibited the synthesis of $TXB_2$ of human peripheral monocytes, one of another mononuclear cell population. A possible explanation of this result is that the increase of $LTB_4$ induced by ionophore may activate the cyclooxygenase in human peripheral monocytes; therefore, this may increase the synthesis of $TXA_2$. LY 255283 may decrease the level of $TXB_2$ in monocytes by inhibiting the synthesis of $LTB_4$.

One can assume that the $LTB_4$ receptor blocker, LY 255283, may also suppress the synthesis of $LTB_4$ by inhibiting 5-LO or $LTA_4$ hydrolase. Such an effect of the compound has not been described previously by other investigators yet and needs further studies using purified 5-LO or $LTA_4$ hydrolase.

In conclusion, besides $LTB_4$'s activation of immunoinflammatory cells like human peripheral PMNL and monocytes, $LTB_4$ can also activate its own synthesis in these cells and activate the synthesis of $TXB_2$ in human monocytes. $LTB_4$ receptor antagonists may provide an additional useful effect by antagonising the self-stimulatory effect of $LTB_4$.

### References

1. Sun FF, McGuire JC. Metabolism of arachidonic acid by human neutrophils. Characterization of the enzymatic reactions that lead to the synthesis of leukotriene $B_4$. *Biochem Biophys Acta* 1984; **794**: 56–65.
2. Goldyne ME, Burrish GF, Poubelle P, Borgeat P. Arachidonic acid metabolism among human mononuclear leukocytes. Lipoxygenase-related pathways. *J Biol Chem* 1984; **259**: 8815–8819.
3. Bray MA, Ford-Hutchinson AW, Smith MJH. Leukotriene $B_4$: An inflammatory mediator *in vivo*. *Prostaglandins* 1981; **22**: 213–222.
4. Rola-Pleszczyniski M, Lemaire I. Leukotrienes augment interleukin-1 production by human monocytes. *J Immunol* 1985; **135**: 3958–3961.
5. Brain SD, Camp RD, Black AB, Wollard PM, Mallet AI, Greaves MV. Psoriasis and leukotriene $B_4$. *Lancet* 1982; **II**: 762.
6. Klickstein LB, Shaplergh C, Goetzl EJ. Lipoxygenation of arachidonic acid as a source of polymorphonuclear leukocyte chemotactic factors in synovial fluid and tissue in rheumatoid arthritis and spondyloarthritis. *J Clin Invest* 1980; **66**: 1166–1170.
7. Snyder DW, Fleisch JH. Leukotriene receptor antagonists as potential therapeutic agents. *Annu Rev Pharmacol Toxicol* 1989; **29**: 123–143.
8. McDonald PP, McColl SR, Naccache PH, Borgeat P. Activation of the human neutrophil 5-lipoxygenase by leukotriene $B_4$. *Br J Pharmacol* 1992; **107**: 226–232.
9. Herron DK, Bollinger NG, Swanson-Bean D, Jackson WT, Froelich LL, Goodson T. LY 255283. A new leukotriene $B_4$ antagonist. *FASEB J* 1988; **2**: A1110 (Abst.)
10. Dewar C. An improved method for isolation of granulocytes from peripheral blood. *J Immunol Methods* 1978; **20**: 301–310.
11. Pradelles P, Grassi J, Maclouf J. Enzyme immunoassay of eicosanoids using AchE as label: an alternative to RIA. *Anal Biochem* 1985; **57**: 1170–1173.
12. Moroney MA, Forder RA, Carey F, Hoult JRS. Differential regulation of 5-lipoxygenase and cyclo-oxygenase pathways of arachidonate metabolism in rat peritoneal leukocytes. *Br J Pharmacol* 1990; **101**: 128–132.
13. Rouzer CA, Kargman S. Translocation of 5-lipoxygenase to the membrane in human leukocytes challenged with ionophore A323187. *J Biol Chem* 1988; **263**: 10980–10988.
14. Elliott GR, Lauwen APM, Bonta IL. Leukotriene $B_4$ stimulation of macrophage cyclooxygenase metabolite synthesis. *Agents Actions* 1991; **32**: 73–74.

ACKNOWLEDGEMENTS. This study was supported by the Turkish Scientific and Technical Research Council (TAG-716) and Ankara University Research Fund (92-09-0019). The authors would like to thank Professor R. K. Türker for his invaluable criticism.

**Received 19 July 1993; accepted 23 August 1993**
